# Assessment of key parameters of normal uterus in women of reproductive age

**DOI:** 10.1038/s41598-023-44489-6

**Published:** 2023-10-18

**Authors:** Fang Chen, Yingxin Gong, Yu Xie, Lei Zhu, Limei Chen, Jingjing Xiao, Ninghong Jiang, Li Sun, Long Sui

**Affiliations:** 1 Medical Center of Diagnosis and Treatment for Cervical Diseases, Obstetrics and Gynecology Hospital of Fudan University, No.419 Fangxie Road, Shanghai, 200011 China; 2 Shanghai Key Laboratory of Female Reproductive Endocrine Related Diseases, Obstetrics and Gynecology Hospital of Fudan University, Shanghai, China; 3 Ultrasound Diagnosis Center, Obstetrics and Gynecology Hospital of Fudan University, No.419 Fangxie Road, Shanghai, 200011 China

**Keywords:** Anatomy, Urogenital diseases

## Abstract

Currently, the precise and detailed anatomical data of the normal uterus, especially the myometrium thickness in various parts of the uterus, are lacking. This study aims to provide normal references for uterine size in healthy reproductive-aged Chinese women to facilitate the application of hysteroscopic surgery. A total of 298 women of reproductive age with normal uterine were included. Parity was significantly correlated with uterine measurements (P < 0.05), and age impacted several measurements (P < 0.05). At each uterine site examined, the myometrium was thinner in nulliparous women than in parous or primiparous women (P < 0.001). Similarly, the extrauterine measurements for parous or primiparous women were larger than those for nulliparous women. Weight affected some external measurements but not myometrial thicknesses, while height did not affect uterine measurements (P > 0.05). There was a positive correlation between body mass index (BMI) and extrauterine measurements as well as myometrial thickness (P < 0.05). The mathematical model of the uterine size for women of reproductive age was constructed stratified by parity. The study is the first to provide a detailed statistical description of the accurate anatomical parameters of the uterus in Chinese reproductive-aged women and has great significance for improving the safety and effectiveness of hysteroscopic surgery for patients.

## Introduction

The uterus is one of the female reproductive organs located internally. It is a hollow organ located in the pelvic cavity's center. The uterus is significantly different in size during adolescence, pregnancy, and disease states^1,2^. For this reason, uterine measurement data is extremely important for monitoring the health of the uterus. As clinicians, we continue to cite the anatomical data of in vitro uterine specimens published by Duffy et al. in 1991 and Holm-Nielsen et al. in 1993 respectively^3,4^. Normal uterus anatomical data is currently lacking.

Not only in outpatient settings but also in uterine surgeries, the normal uterus parameter is crucial. For instance, the diagnosis of the uterine incomplete septum is determined by comparing the thickness of the uterine fundus myometrium of a patient with a normal uterus. In addition to uterine metroplasty, hysteroscopic surgery will depend on the uterus's anatomical parameters. Therefore, the establishment of anatomical data for the uterus of women of childbearing age is essential for standardizing accurate and safe uterine septum metroplasty, and it is also a key clinical problem that many gynecologists have been attempting to solve for decades. Nonetheless, there was no adequate sample to serve as a standard for a normal uterine size for surgical procedures.

In this study, we aim to establish a comprehensive mathematical model of the in vivo uterus and provide more detailed uterine parameters in normal, healthy women and correlate them with chronological age, height, weight, body mass index (BMI), and parity in these normal subjects. This epidemiology data will provide obstetricians and gynecologists with sufficient evidence for patient physical examination, as well as a theoretical basis and surgical guidelines for uterine surgery.

## Materials and methods

### Population selection and data collection

This prospective survey study was performed on healthy women aged 18 to 47 years who came to Obstetrics and Gynecology Hospital of Fudan University for an annual checkup from October 2018 to August 2020. Women presenting with fibroids, adenomyosis, malignant tumors, pregnancy, incomplete abortion, uterine anomalies, metrauxe and a history of abortion were excluded, as well as those with incomplete medical records. Subjects with dysmenorrhea, chronic pelvic pain, menorrhagia, and any history of uterine surgery also did not meet the enrollment criteria for this study. A total of 298 participants who were found to be normal on clinical and anthropometric assessment were enrolled. The research protocol was reviewed and approved by the institutional ethics review board of the Obstetrics and Gynecology Hospital of Fudan University, and all methods were performed in accordance with the relevant guidelines and regulations. Signed informed consent documents were collected from all participants.

Data on the clinical characteristics of all women in the study were collected, including age, height, weight, parity, BMI, and reproductive history. BMI was calculated as weight in kilograms over height in meters squared (kg/m^2^) based on data documented in the participant’s registration information. BMI was categorized based on the World Health Organization classification into three groups: underweight (< 18.5 kg/m^2^), normal weight (18.5–24.9 kg/m^2^) and overweight (≥ 25 kg/m^2^)^5^. The cohort was then divided into different groups based on these factors.

### Uterine measurement

**Figure 1:**
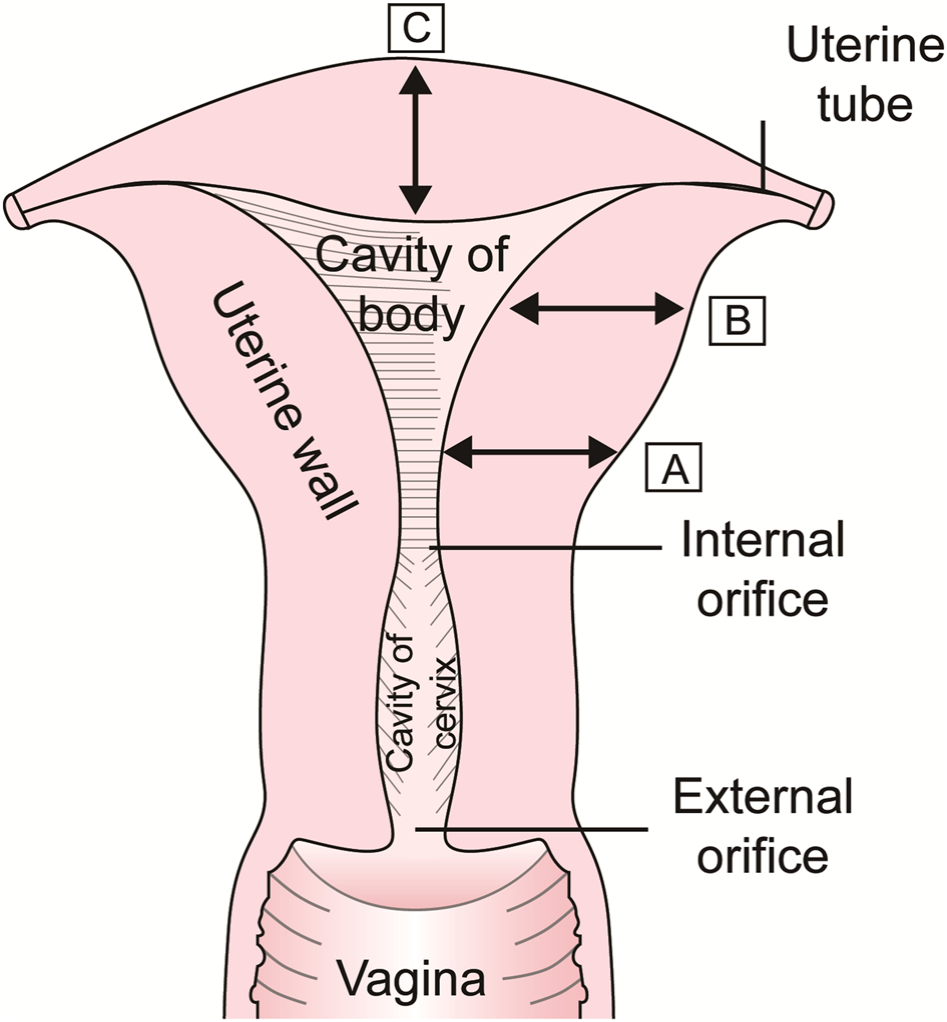
Anatomical diagram of the uterus and the sagittal and transverse sections of the uterus used to measure wall thickness. A, Anterior lower segment; B, Mid-anterior wall; C, fundal wall.

**Figure 2:**
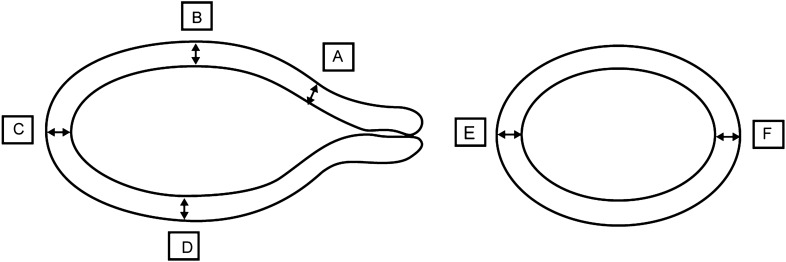
Sagittal and transverse sections of the uterus used for the measurement of myometrial thickness. A, Anterior lower segment; B, Mid-anterior wall; C, fundal wall; D, posterior wall; E, right wall; F, left wall.

Ultrasonography (US) is a noninvasive and useful measuring tool for female reproductive organ imaging^6^_._ Transvaginal or transrectal ultrasound was performed using a scanner with a 5 to 9-MHz intraluminal probe (PHILIP HD11-XE) by an experienced sonographer. In each patient, the length of the uterine body(the distance from the middle point of the serous membrane at the bottom of the uterus to the internal cervical orifice), length of the cervix (the distance from the internal orifice to the external orifice), depth of the uterine cavity (the distance from the middle point of the mucous membrane at the bottom of the uterus to the internal cervical orifice), Body anteroposterior diameter (the maximum anteroposterior distance perpendicular to the length of the uterine body), Body transverse diameter (the maximum transverse diameter of the uterus measured slightly below the cornua of the uterus) and myometrial thickness(MT) at various parts of the uterus were measured. The markers of the uterine measurement sites were shown in Figs. 1 and 2.

The uterus is shaped like a prolate ellipsoid, and its spatial location in the pelvis changes as the bladder fills, which alters the two-dimensional lines of it. However, the change in uterine volume is small. To reduce error and bias, all subjects emptied their bladders prior to the ultrasound examination. The protocol for this study was approved by the ethics committee of our university, and informed consent was obtained from the participants.

### Statistical analysis

Data are presented as the mean ± standard deviation (SD) of each group. The mean values of the variables with normal distributions for different items were compared by ANOVA, and Student’s t-test was used to determine differences in myometrial thickness at each uterine site. Spearman rank correlation was used to analyze the relationship between the parity and uterine parameters and Pearson’s linear correlation was used to analyze the relationships between the abovementioned variables and other uterine parameters, and P < 0.05 was considered significant. SPSS for Windows version 21.0 was used for the analysis.

## Results

### Demographic characteristics of the population

A total of 298 healthy women were enrolled in this study. There were 189 (63.43%) nulliparous women and 109 (36.57%) parous women, among whom 76 had 1 birth and 33 had ≥ 2 births. The mean age of these women was 28.82 ± 5.31 years, with a range of 18 to 47 years. The mean height of the population was 161 ± 4.37 cm (range:150–170 cm), and their average presenting weight was 54.5 ± 8.17 kg (range: 40–80 kg). Considering the comprehensive evaluation of height and weight, the body max index (BMI) was calculated. Among the whole population, 72.15% (215/298) of the women was in normal weight, 12.08% (36/298) of the population was underweight and the rest 15.77% (47/298) was overweight.

### Multivariate analysis about parity, age, weight, BMI, height and uterine measurements

In order to clarify the independent factor of uterine measurements, multivariate analysis about parity, age, weight, BMI and height was conducted. As depicted in Table 1, the results of the analysis suggested that parity was correlated with all uterine measurements (P < 0.05) except for cervical length (P = 0.120), and age impacted several measurements including body anteroposterior diameter, cervical length, the myometrial thickness of the fundal wall, mid-anterior wall, anterior lower segment and lower side wall (P < 0.05). Weight affected some external measurements including body length (P = 0.021), cervical length (P < 0.001) and uterine depth (P = 0.043) but not myometrial thicknesses (P > 0.05), while height did not affect uterine measurements (P > 0.05). BMI only correlated with body transverse diameter (P = 0.032).

**Table 1:** Multivariate analysis about parity, age, weight, BMI, height, extrauterine measurements and myometrial thickness at different uterine sites.

	Coefficients (B)	Std. Error	T-statistic	P-value
Extrauterine measurements				
Body L	39.133	2.558	15.299	< 0.001
Parity	2.121	0.423	5.015	** < 0.001**
Age			1.500	0.135
Height			− 0.204	0.839
Weight	0.108	0.046	2.334	**0.021**
BMI			0.208	0.835
Body Td	37.061	2.965	12.498	< 0.001
Parity	2.654	0.445	5.963	** < 0.001**
Age			1.367	0.173
Height			0.236	0.813
Weight			0.282	0.778
BMI	0.304	0.140	2.164	**0.032**
Body Ad	29.721	2.330	12.756	< 0.001
Parity	1.707	0.447	3.819	** < 0.001**
Age	0.237	0.082	2.878	**0.004**
Height			0.145	0.885
Weight			1.514	0.132
BMI			1.643	0.102
Cervical L	20.064	1.920	10.448	< 0.001
Parity			1.560	0.120
Age	0.149	0.047	3.181	**0.002**
Height			− 0.442	0.659
Weight	0.131	0.028	4.690	** < 0.001**
BMI			0.576	0.565
Depth of Uc	29.908	2.277	13.133	< 0.001
Parity	1.530	0.376	4.065	** < 0.001**
Age			0.669	0.504
Height			0.124	0.901
Weight	0.084	0.041	2.036	**0.043**
BMI			− 0.120	0.905
Myometrial thickness				
Fundal wall	8.330	0.955	8.726	< 0.001
Parity	0.443	0.183	2.420	**0.016**
Age	0.079	0.034	2.353	**0.020**
Height			− 0.176	0.861
Weight			1.046	0.297
BMI			1.213	0.227
Mid-anterior wall	8.977	1.135	7.913	< 0.001
Parity	1.069	0.218	4.910	** < 0.001**
Age	0.119	0.040	2.965	**0.003**
Height			0.498	0.619
Weight			1.867	0.063
BMI			1.882	0.061
Mid-side wall	12.520	0.166	75.321	< 0.001
Parity	0.972	0.171	5.700	** < 0.001**
Age			0.607	0.545
Height			1.048	0.296
Weight			1.820	0.070
BMI			1.502	0.135
Anterior lower segment	4.859	0.715	6.798	< 0.001
Parity	0.742	0.137	5.409	** < 0.001**
Age	0.105	0.025	4.155	** < 0.001**
Height			− 0.504	0.614
Weight			0.781	0.436
BMI			1.087	0.279
Lower side wall	5.844	0.838	6.974	< 0.001
Parity	0.648	0.161	4.032	** < 0.001**
Age	0.075	0.030	2.526	**0.012**
Height			1.240	0.217
Weight			1.862	0.064
BMI			1.495	0.137
Left cornua uteri	3.648	0.044	82.582	< 0.001
Parity	0.279	0.045	6.155	** < 0.001**
Age			− 0.857	0.393
Height			− 0.367	0.714
Weight			1.453	0.148
BMI			1.714	0.088
Right cornua uteri	3.633	0.045	81.136	< 0.001
Parity	0.287	0.046	6.250	** < 0.001**
Age			− 0.394	0.694
Height			− 0.505	0.614
Weight			1.367	0.173
BMI			1.665	0.098

L, length; Td, transverse diameter; Ad, anteroposterior diameter; Uc, uterine cavity.

Significant values are in bold.

### Correlation between uterine measurements and parity

**Figure 3:**
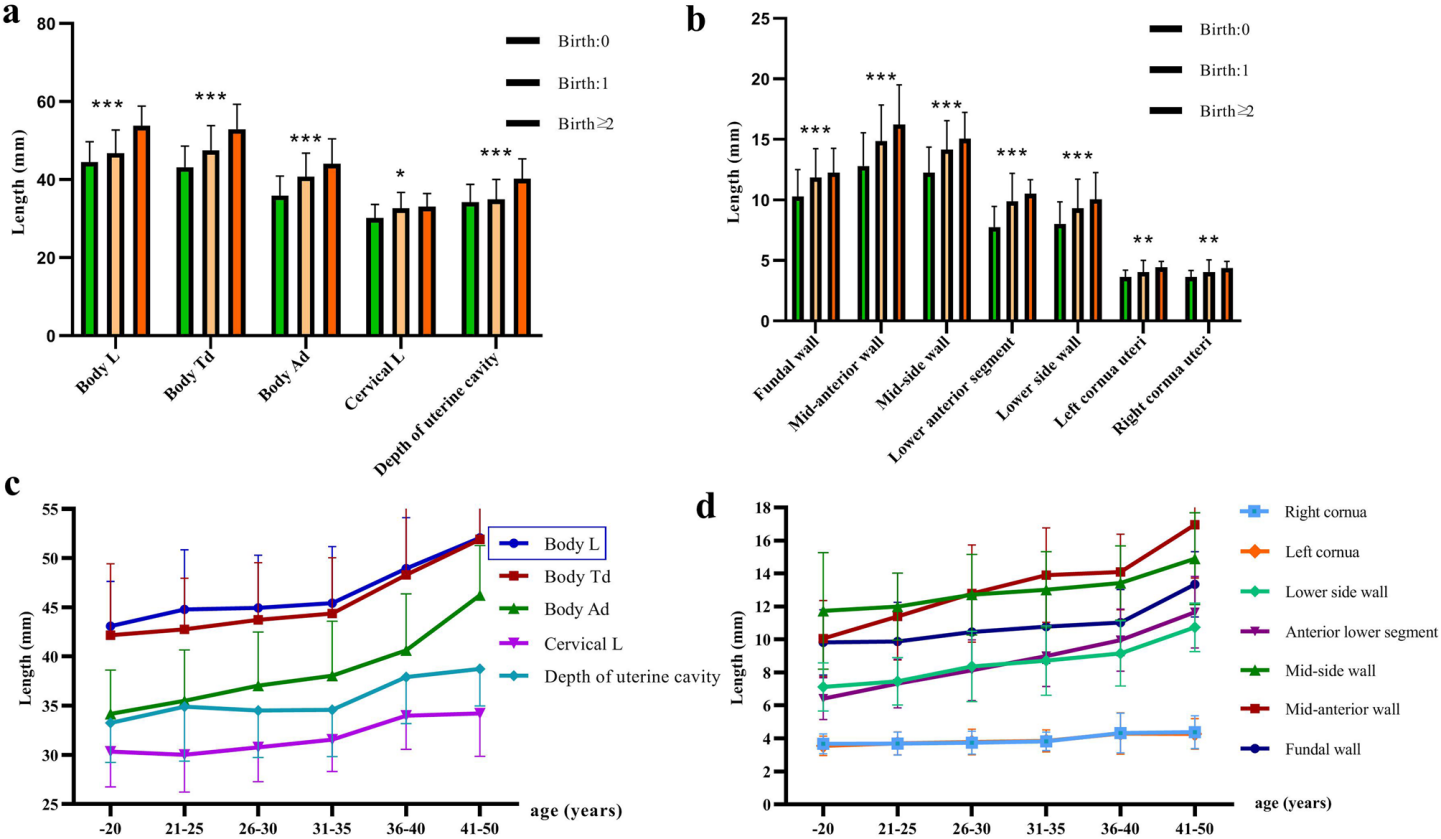
(**a**) Correlation between extrauterine measurements and parity. (**b**) Correlation between myometrial thickness at different uterine sites and parity. (**c**) Correlation between extrauterine measurements and chronological age. (**d**) Correlation between myometrial thickness at different uterine sites and chronological age.

As shown in Table 2, the study population was further divided into three groups for correlation analysis based on parity. Compared to nulliparas, the parameters including various extrauterine measurements and the myometrial thicknesses at different uterine sites were all significantly higher in parous women (P < 0.001). Moreover, as the number of deliveries increased, the measurements of all parameters increased significantly (r > 0.3, P < 0.001). Figure 3a,b illustrated these trends more intuitively and clearly showed the positive correlation between the uterine parameters and parity. Moreover, the myometrial thickness of the mid-anterior wall and mid-side wall was the highest of the parameters, followed by the thickness of the fundus of the uterus, and the myometrial thickness of the cornua uteri was the lowest of the parameters.

**Table 2:** Extrauterine measurements and myometrial thickness at different uterine site by parity.

Parameters	Parity			Spearman rank correlation	
	0 (N = 189)	1 (N = 76)	≥ 2 (N = 33)	P-value	r
Extrauterine measurements (mm)					
Body L	44.49 ± 5.20	46.77 ± 5.92	53.75 ± 5.06	< 0.001	0.373
Body Td	43.14 ± 5.41	47.47 ± 6.37	52.87 ± 6.40	< 0.001	0.363
Body Ad	35.88 ± 5.00	40.74 ± 6.04	44.02 ± 6.43	< 0.001	0.374
Cervical L	30.15 ± 3.48	32.60 ± 4.07	33.00 ± 3.41	0.002	0.396
Depth of Uc	34.22 ± 4.52	34.90 ± 5.14	40.23 ± 5.08	0.007	0.384
Myometrial thickness (mm)					
Fundal wall	10.28 ± 2.23	11.87 ± 2.37	12.26 ± 2.01	< 0.001	0.398
Mid-anterior wall	12.80 ± 2.75	14.86 ± 2.99	16.23 ± 3.27	< 0.001	0.395
Mid-side wall	12.27 ± 2.09	14.15 ± 2.41	15.06 ± 2.17	< 0.001	0.381
Anterior lower segment	7.75 ± 1.72	9.88 ± 2.32	10.51 ± 1.17	< 0.001	0.460
Lower side wall	8.03 ± 1.82	9.30 ± 2.41	10.05 ± 2.20	< 0.001	0.364
Left cornua uteri	3.64 ± 0.56	4.03 ± 0.99	4.43 ± 0.50	< 0.001	0.420
Right cornua uteri	3.64 ± 0.54	4.05 ± 1.01	4.38 ± 0.54	< 0.001	0.397

N, number of subjects; L, length; Td, transverse diameter; Ad, anteroposterior diameter; Uc, uterine cavity.

This indicated that the size of the uterus in parous women was larger than that in nulliparous women. All anatomical parameters increased significantly with the increasing number of parity, and the difference was significant (Table 2 and Fig. 3a,b), which indicates that parity influences the morphology of the uterus.

### Correlation between uterine measurements and chronological age

As Table 3 indicated, all the uterine parameters for extrauterine measurements increased with chronological age. A significant positive linear relationship was observed between myometrial thickness at each uterine site and age (r > 0.3, P < 0.001). The increasing trend in the extrauterine measurements was particularly significant between the ages of 31 and 50 years (P < 0.001, Fig. 3c). Moreover, the increasing trend of the myometrial thickness of uterine sites apart from cornua uteri was even more remarkable between the ages of 36 and 50 years (P < 0.001, Fig. 3d). Compared with other uterine sites, the myometrial thickness in cornua uteri was the thinnest and remained relatively stable with age. However, in order to exclude the influence of pregnancy on uterine size and myometrium thickness, and further explore the influence of single factor age on extrauterine measurement and myometrium thickness, we excluded women who had given birth and conducted independent analysis on women who had not given birth. It can be seen from Table 4 that with the increase of age, the extrauterine measurement diameter showed an overall trend of increasing, but P < 0.05. The difference was not statistically significant. In addition to the lower uterine muscle thickness being positively correlated with age (P < 0.05), the other parts of the muscle thickness were not significantly positively correlated with age, and the difference was not statistically significant. This indicates that the trend of increase in extrauterine measurements and muscular thickness as shown in Table 3 with age may be mainly influenced by the number of pregnancies, and the older the age, the more pregnancies may be.

**Table 3:** Extrauterine measurements and myometrial thickness at different uterine sites by chronological age.

Parameters	Age(years)						Pearson line correlation	
	-20 (N = 15)	21–25 (N = 66)	26–30 (N = 117)	31–35 (N = 63)	36–40 (N = 21)	41–50 (N = 16)	P-value	r
Extrauterine measurements (mm)								
Body L	43.08 ± 4.56	44.80 ± 6.06	44.95 ± 5.35	45.43 ± 5.74	48.94 ± 5.18	52.08 ± 5.17	< 0.001	0.383
Body Td	42.17 ± 7.25	42.76 ± 5.21	43.73 ± 5.81	44.37 ± 5.69	48.31 ± 7.00	51.92 ± 5.16	< 0.001	0.329
Body Ad	34.17 ± 4.47	35.50 ± 5.18	37.05 ± 5.45	38.05 ± 5.55	40.63 ± 5.75	46.23 ± 5.07	< 0.001	0.376
Cervical L	30.33 ± 3.58	30.03 ± 3.81	30.78 ± 3.50	31.56 ± 3.23	34.00 ± 3.42	34.23 ± 4.38	< 0.001	0.393
Depth of Uc	33.25 ± 4.01	34.92 ± 5.54	34.52 ± 4.78	34.60 ± 4.76	37.91 ± 4.73	38.74 ± 3.77	0.007	0.394
Myometrial thickness (mm)								
Fundal wall	9.83 ± 1.98	9.88 ± 2.38	10.45 ± 2.26	10.78 ± 2.23	11.02 ± 2.01	13.34 ± 1.98	< 0.001	0.397
Mid-anterior wall	10.06 ± 2.31	11.39 ± 2.63	12.78 ± 2.95	13.9 ± 2.86	14.09 ± 2.3	16.95 ± 3.22	< 0.001	0.427
Mid-side wall	11.73 ± 3.54	11.99 ± 2.03	12.71 ± 2.44	13.01 ± 2.31	13.43 ± 2.26	14.9 ± 2.79	0.001	0.456
Anterior lower segment	6.41 ± 1.27	7.33 ± 1.48	8.13 ± 1.84	8.99 ± 1.85	9.96 ± 1.88	11.65 ± 2.17	< 0.001	0.496
Lower side wall	7.12 ± 1.46	7.46 ± 1.44	8.36 ± 2.13	8.72 ± 2.1	9.16 ± 1.98	10.74 ± 1.47	< 0.001	0.371
Left cornua uteri	3.55 ± 0.59	3.70 ± 0.70	3.78 ± 0.78	3.85 ± 0.67	4.3 ± 1.25	4.27 ± 0.93	0.016	0.426
Right cornua uteri	3.68 ± 0.65	3.69 ± 0.72	3.74 ± 0.75	3.82 ± 0.56	4.33 ± 1.23	4.38 ± 1.00	0.005	0.389

Abbreviations: N, number of subjects; L, length; Td, transverse diameter; Ad, anteroposterior diameter; Uc, uterine cavity.

**Table 4:** Extrauterine measurements and myometrial thickness at different uterine sites by chronological age of nulliparous women.

Parameters	Age (years) (nulliparous)					Pearson line correlation	
	-20 (N = 11)	21–25 (N = 49)	26–30 (N = 85)	31–35 (N = 36)	36–40 (N = 7)	P-value	r
Extrauterine measurements (mm)							
Body L	43.27 ± 4.73	45.00 ± 5.77	43.94 ± 4.81	45.28 ± 5.53	46.00 ± 4.69	0.247	0.085
Body Td	42.36 ± 7.57	43.02 ± 4.99	42.81 ± 5.18	43.94 ± 5.93	44.86 ± 5.64	0.296	0.077
Body Ad	34.09 ± 4.68	35.24 ± 5.00	35.76 ± 4.74	37.08 ± 5.77	38.14 ± 3.85	0.061	0.137
Cervical L	30.73 ± 3.47	30.45 ± 3.99	30.65 ± 3.24	31.28 ± 3.32	32.43 ± 2.51	0.104	0.119
Depth of Uc	33.27 ± 4.21	34.69 ± 4.99	33.78 ± 4.40	34.68 ± 4.39	35.37 ± 3.71	0.485	0.051
Myometrial thickness (mm)							
Fundal wall	10.00 ± 1.98	10.31 ± 2.42	10.16 ± 2.14	10.60 ± 2.35	10.63 ± 1.60	0.235	0.087
Mid-anterior wall	10.04 ± 2.42	11.47 ± 2.41	12.26 ± 2.53	13.65 ± 3.14	12.73 ± 1.83	< 0.001	0.271
Mid-side wall	11.76 ± 3.71	12.30 ± 1.97	12.33 ± 2.01	12.32 ± 1.99	11.73 ± 1.64	0.788	0.020
Anterior lower segment	6.25 ± 1.20	7.31 ± 1.43	7.76 ± 1.63	8.54 ± 1.99	8.84 ± 1.52	< 0.001	0.318
Lower side wall	7.05 ± 1.51	7.52 ± 1.45	8.07 ± 1.70	8.79 ± 2.36	8.54 ± 1.64	< 0.001	0.264
Left cornua uteri	3.45 ± 0.51	3.65 ± 0.68	3.61 ± 0.48	3.74 ± 0.56	3.61 ± 0.59	0.893	− 0.010
Right cornua uteri	3.52 ± 0.32	3.64 ± 0.69	3.61 ± 0.48	3.74 ± 0.51	3.61 ± 0.60	0.999	− 0.010

N, number of subjects BMI, body mass index.

### Correlation between uterine measurements and BMI

Although multivariate analysis only showed the correlation between BMI and body transverse diameter (P = 0.032, Table 1). We further categorized BMI into three groups: underweight (< 18.5 kg/m^2^), normal weight (18.5–24.9 kg/m^2^) and overweight (≥ 25 kg/m^2^). Table 5 revealed a positive correlation between body mass index (BMI) and extrauterine measurements as well as myometrial thickness (P < 0.05). When further analyzing the myometrial thickness at different uterine sites, the myometrial thickness of the mid-anterior wall and mid-side wall was the highest of the parameters, followed by the thickness of the fundus of the uterus, and the myometrial thickness of the cornua uteri was the lowest of the parameters.

**Table 5:** Extrauterine measurements and myometrial thickness at different uterine sites by BMI.

Parameters	BMI						Pearson line correlation	
	Underweight, < 18.5 (N = 36)		Normal weight , 18.5–24.99 (N = 215)		Overweight, ≥ 25 (N = 47)		P-value	r
	Mean ± SD	95% CI	Mean ± SD	95% CI	Mean ± SD	95% CI		
Extrauterine measurements (mm)								
Body L	43.76 ± 4.96	42.03–45.50	46.09 ± 5.64	45.17–47.00	49.00 ± 7.19	45.02–52.98	0.016	0.170
Body Td	42.09 ± 6.09	39.96–44.22	44.75 ± 6.03	43.77–43.73	47.20 ± 6.56	43.56–50.83	0.015	0.168
Body Ad	35.21 ± 4.73	33.56–36.86	37.38 ± 5.75	36.44–38.31	39.60 ± 6.88	35.78–43.41	0.032	0.151
Myometrial thickness (mm)								
Fundal wall	10.62 ± 2.18	9.86–11.38	10.73 ± 2.28	10.36–11.10	11.76 ± 2.27	10.50–13.01	0.020	0.116
Mid-anterior wall	11.74 ± 2.47	10.87–12.60	12.95 ± 2.78	12.50–13.40	13.89 ± 3.97	11.69–16.09	0.018	0.166
Mid-side wall	12.10 ± 2.35	11.28–12.91	12.99 ± 2.45	12.62–13.36	13.51 ± 2.67	12.03–14.99	0.041	0.122
Anterior lower segment	7.64 ± 1.64	7.06–8.21	8.26 ± 1.85	7.96–8.56	8.51 ± 2.59	7.07–9.94	0.033	0.125
Lower side wall	7.32 ± 1.17	6.91–7.73	8.46 ± 2.11	8.11–8.79	8.07 ± 1.95	6.99–9.16	0.011	0.139
Left cornua uteri	3.59 ± 0.49	3.42–3.76	3.78 ± 0.67	3.67–3.89	3.91 ± 0.44	3.67–4.16	0.013	0.139
Right cornua uteri	3.60 ± 0.51	3.42–3.78	3.76 ± 0.68	3.65–3.87	3.88 ± 0.45	3.63–4.13	0.042	0.133

N, number of subjects BMI, body mass index.

### Construction of the mathematical model of the uterine size for women of reproductive age

According to the above results, we could obtain the uterine parameters of reproductive-aged women in Shanghai for external measurements and myometrial thickness. The above findings indicated that after delivery, the external measurement and myometrial thickness of the uterus increased significantly. The statistical analysis showed significant differences between nulliparas and paras. Thus, the mathematical model of the uterine size was constructed under the stratified nulliparous and parous women, as shown in Table 6.

**Table 6:** The mathematical model of the uterine size for women of reproductive age in Shanghai.

Parameters	Nulliparas			Multiparas		
	Mean ± SD (mm)	95% CI (mm)	Min–max (mm)	Mean ± SD (mm)	95% CI (mm)	Min–max (mm)
Body L	44.49 ± 5.20	43.74–45.23	34–63	48.14 ± 6.50	46.43–49.85	33–63
Transverse diameter	43.14 ± 5.41	42.36–43.92	29–58	48.31 ± 6.63	46.57–50.05	34–68
Anteroposterior diameter	35.88 ± 5.00	35.16–36.60	24–53	41.29 ± 6.01	39.71–42.87	30–55
Cervical L	30.75 ± 3.48	30.25–31.25	22–40	32.64 ± 4.00	31.58–33.69	23–42
Depth of uterine cavity	34.22 ± 4.52	33.57–34.87	25–50	36.21 ± 5.84	34.67–37.74	21.6–52.1
Fundal wall	10.28 ± 2.23	9.96–10.60	4.1–15.80	11.93 ± 2.23	11.34–12.52	5.8–18
Mid-anterior wall	12.17 ± 2.76	11.79–12.58	5.8–22.1	15.21 ± 3.18	14.38–16.05	8.9–23
Mid-side wall	12.29 ± 2.08	11.99–12.59	5.6–17.5	14.43 ± 2.41	13.79–15.06	8.5–20
Anterior lower segment	7.75 ± 1.72	7.50–7.80	4.4–14.3	10.06 ± 2.13	9.50–10.62	4.8–15.1
Lower side wall	8.03 ± 1.83	7.77–8.30	4.3–14.7	9.56 ± 2.40	8.92–10.19	6.1–20
Left cornua uteri	3.64 ± 0.56	3.56–3.72	1.7–5.3	4.15 ± 0.94	3.90–4.40	2.1–8.2
Right cornua uteri	3.64 ± 0.54	3.57–3.72	1.7–5.2	4.16 ± 0.96	3.91–4.41	2.3–8.2

N, number of subjects; L, length.

## Discussion

In this study, we demonstrate the significance and clinical value of the anatomical parameters of the uterus of healthy women. To our knowledge, this is the very first study to provide specific data regarding the uterus anatomy of healthy Chinese women.

In recent years, there have been few reports on relevant measurements of the normal uterus in healthy women of reproductive age. In general, uterine measurement data varied with race and region^7^. In 1991, Duffy reported 20 cases of an in vitro uterus, and the results showed that the fundus of the uterus had an average thickness of 1.4 cm, the anterior wall had an average thickness of 1.8 cm, and the back wall had an average thickness of 1.9 cm. However, the uterine isthmus was 1.3 cm (the thinnest point was only 7 mm). The thickness of cornua uteri which were 5 mm from the oviduct openings was 6 mm (the thinnest point was only 4 mm)^3^. In 1993, Holm-Nielsen et al. reported 8 cases of an in vitro uterus, and the results showed that the average thicknesses of the front and back walls were more than 2 cm. The thickness of the fundus was 0.95–1 cm, and the thickness of the uterine isthmus was only half the thickness of the corpus (1.0 cm). The thickness of the cornua uteri was 6 mm (the thinnest point was only 4 mm)^4^. The above data were not corrected to the shrinkage rate (45%) when the uterus was in vitro. If the data were adjusted to the shrinkage rate, it would not be difficult to determine that the myometrial thickness of European and American women is generally larger than that of Asian ethnic groups. However, when comparisons are made within an Asian population, the myometrial thicknesses of the anterior and back walls are larger than that of the cornua uteri^8^. This finding is consistent with the results of this study, namely, that the myometrial thickness of the cornua uteri is the thinnest part of the uterus. The main cause is its own anatomical characteristics. In their study, the data indicated that the myometrial thickness of European and American women is generally larger than that of Asians.

The uterus is shaped like a prolate ellipsoid with myometrial thickness varying from different planes. Thus, the myometrial thickness of the middle uterine segment is larger than that of the lower uterine segment. However, the myometrial thickness of the uterus on the same horizontal plane is identical, which has been demonstrated in this study, such as the thickness of the mid-anterior wall is approximately equal to that of the mid-side wall and the thickness of the left cornua uteri is approximately equal to that of the right cornua uteri of the uterus.

This study attempted to find the factors that might contribute to the changes in uterine measurements. The results demonstrated that the uterine parameters significantly increased with age and parity. The finding is in accordance with previous studies, which indicate that uterine size is affected by age and parity in both productive age and postmenopausal women^9,10^_._ This finding could be explained by the fact that as the number of births increases, the more the uterine muscle fibers become stretched when the uterus expands, and the muscle cells become hypertrophic, leading to an increase in muscle layer thickness^11,12^.

According to the statistics, the ratio of the uterine body length to the cervical length was fixed at 1.5:1, regardless of parity. Our data further showed that the increments in the uterine parameters, such as myometrial thickness, body length, transverse diameter, and the anteroposterior dimensions of the body, were extremely obvious between 36- and 50-year-old women. The correlation between uterine size and BMI is still controversial at present. Although a positive correlation between BMI and uterine size independent of age and parity is proven by some studies, most studies do not recognize the correlation^10,13,14^. However, our study revealed a positive correlation between uterine parameters and BMI, while no significant correlation with height and weight was spotted. Albeit significantly correlated, the association needs to be further assessed as the rank correlation coefficient was low, which suggested a weak correlation.

This study aimed to provide anatomical data of the normal uterus for women of reproductive age and to build a mathematical model that could be used to guide clinical applications. Take the management of the septate uterus as an application. With an incidence of 0.01–12%, the uterus septum is associated with a higher rate of recurrent miscarriages, reproductive failure and obstetric complications^15^. With the increasing prevalence of hysteroscopic surgeries and continuous improvement in relevant equipment and instruments, a growing number of women with a uterine septum have been successfully treated via metroplasty with the benefit of avoiding laparotomy and even hospitalization over the past 20 years^16,17^. During the operation of transcervical resection of the septum (TCIS), the uterine septum can be cut up to 1.0–1.5 cm but not less than 1.0 cm from the uterine fundus membrane under ultrasound guidance, which greatly improves the operation efficacy^18,19^. Our study presents a mathematical model of the uterine size for women of childbearing age, which can provide an objective basis for the termination criteria for hysteroscopic surgery. When the depth of resection is insufficient, the recurrence rates of the uterine septum and related symptoms increase. In hysteroscopic surgery, efforts should be made to bring the rest of the myometrium as close to the normal model as possible in terms of the uterine structure. On the contrary, excessive surgical removal of mediastinal tissue can easily lead to uterine perforation, peripheral viscera injury and other serious complications, especially on the side of the uterine horn and uterine isthmus, in which the myometrial tissue is weak. Whether perforation occurs or not closely relates to the thickness of the uterine wall. Our findings showed that the myometrial thickness was almost the same in the anterior and posterior walls of the uterus in the same cross-section, while the myometrial thickness in the middle segment of the uterus was larger than that in the lower uterine segment. Furthermore, the myometrial thickness at the cornua uteri was the thinnest, with a mean thickness of 3.8 mm, while the fundus wall was an average of approximately 10.2 mm in nulliparous women and 11 mm in parous women. During hysteroscopic surgery, more attention must be paid to the fundus of the uterus to avoid excessive surgical removal of tissue or electric coagulation for a long time to avoid the occurrence of intraoperative and postoperative complications. From this research, and in combination with our previous studies, we determined the endpoint criteria for TCIS treatment^17,20^. In addition, in the treatment of endometrial polyps, intrauterine adhesions, uterine cesarean section scar diverticula and other related diseases by hysteroscopic surgery, proficiency with data regarding the normal uterine anatomy is also required.

In conclusion, our study revealed several contributing factors for uterine parameters and constructed the mathematical model of the uterine size for women of reproductive age based on the in vivo ultrasound measurement of key parameters of the normal uterus in the largest Ob/Gyn center in China. This study provides authentic clinical data from healthy women, which may greatly boost the precision and further optimization of hysteroscopic surgery.

## Data Availability

The datasets generated during and/or analyzed during the current study are available from the corresponding author on reasonable request.
